# Parathyroid Carcinoma in a Patient With End-Stage Renal Disease Initially Suspected With Refractory Secondary Hyperparathyroidism

**DOI:** 10.7759/cureus.94267

**Published:** 2025-10-10

**Authors:** Tomohiro Tsuboya, Takeo Ozawa, Tomoaki Asamori, Shotaro Naito, Soichiro Iimori

**Affiliations:** 1 Nephrology, Institute of Science Tokyo, Tokyo, JPN; 2 Head and Neck Surgery, Institute of Science Tokyo, Tokyo, JPN

**Keywords:** dialysis, hemodialysis, hypercalcemia, parathyroid carcinoma, parathyroid hormone, secondary hyperparathyroidism

## Abstract

A 50-year-old woman, who developed end-stage renal failure due to immunoglobulin A (IgA) nephropathy, had been on hemodialysis for seven years. One year ago, she underwent evaluation at another hospital for elevated parathyroid hormone (PTH) levels; however, no definitive diagnosis was made, and she was placed under observation. Because secondary hyperparathyroidism (SHPT) was considered the most likely cause in a dialysis patient, her medications were adjusted, but her PTH levels did not improve, and she was referred to our department. Further evaluation revealed a suspicion of parathyroid carcinoma, and parathyroidectomy was performed. Histopathological examination confirmed the diagnosis of parathyroid carcinoma. Postoperatively, her PTH levels normalized promptly. Although secondary hyperparathyroidism is a common cause of hyperparathyroidism in maintenance dialysis patients, this case suggests the importance of considering parathyroid carcinoma as a possible underlying cause.

## Introduction

Parathyroid hormone (PTH) levels start to increase with the progression of chronic kidney disease (CKD) when estimated glomerular filtration rate (eGFR) falls to approximately 45 mL/minute/1.73 m^2^ [1]. At the initiation of maintenance dialysis therapy, nearly all patients present with secondary hyperparathyroidism (SHPT), characterized by persistently elevated PTH levels (normal range: <65 pg/mL), with more than 80% of patients exhibiting serum PTH levels exceeding 150 pg/mL [2]. Therefore, regular monitoring of PTH levels is recommended in dialysis patients, and therapeutic intervention is warranted if target levels are not achieved [3].

In this case, a detailed evaluation was performed for refractory hyperparathyroidism in a patient undergoing hemodialysis, ultimately leading to a diagnosis of parathyroid carcinoma rather than secondary hyperparathyroidism. Furthermore, parathyroidectomy resulted in improvement of bone mineral metabolism.

The prevalence of SHPT in dialysis patients has been reported to range from 30% to 68.6% [4,5]. Given the high prevalence of SHPT in maintenance hemodialysis patients, the diagnosis of parathyroid carcinoma remains uncommon. This case highlights the necessity of considering parathyroid carcinoma in the differential diagnosis of secondary hyperparathyroidism in this population.

## Case presentation

A 50-year-old woman had been initiated on hemodialysis seven years earlier for end-stage renal disease secondary to immunoglobulin A (IgA) nephropathy. Since then, she had been maintained on regular hemodialysis at another facility. One year prior to presentation, she was referred to the endocrinology department of another hospital for evaluation of secondary hyperparathyroidism. At this stage, no overt symptoms were observed, and abnormalities were detected solely through routine laboratory examinations. Cervical ultrasonography at that time revealed no apparent parathyroid gland enlargement, and surgical treatment was considered not feasible. Despite subsequent administration of the maximum dose of etelcalcetide hydrochloride, her intact parathyroid hormone (iPTH) levels remained persistently elevated, ranging from 1,700 to 2,300 pg/mL (normal range: 10-65 pg/mL). She was therefore referred to our department for further evaluation and management.

Her past medical history included diabetes mellitus, diagnosed 10 years earlier, and hypertension of unknown onset. Family history was unremarkable. She was a never-smoker, reported occasional alcohol consumption, and had no known allergies.

At our outpatient clinic, based on the findings of the previous cervical ultrasonography, which revealed no apparent parathyroid gland enlargement, and the persistent marked elevation of iPTH levels despite adequate medical therapy, ectopic hyperparathyroidism was suspected. Technetium-99m methoxyisobutylisonitrile (Tc-MIBI) scintigraphy demonstrated a nodular area with delayed tracer washout in the left thyroid lobe, raising suspicion for a parathyroid tumor. Following consultation with the otolaryngology department, malignancy could not be excluded, and surgical intervention was planned. The patient was admitted to our hospital for parathyroidectomy.

On admission, her oral medications and daily dosages were as follows: ferric citrate hydrate 750 mg, precipitated calcium carbonate 1,500 mg, tenapanor hydrochloride 40 mg, linaclotide, nifedipine, teneligliptin hydrobromide hydrate, febuxostat, polaprezinc, telmisartan, and carvedilol. Medications administered during hemodialysis sessions included etelcalcetide hydrochloride 7.5 mg at each dialysis session and darbepoetin alfa.

On physical examination at admission, her body temperature was 36.5°C, pulse rate 72 beats/minute, blood pressure 185/85 mmHg, respiratory rate 14 breaths/minute, and oxygen saturation 97% on room air. A firm mass measuring slightly less than 3 cm was palpable in the left thyroid lobe region. No other remarkable findings were noted on physical examination.

Laboratory data on admission are summarized in Table 1.

**Table 1:** Preoperative blood tests AST: aspartate transaminase, ALT: alanine transaminase, γ-GT: gamma-glutamyl transferase, CRP: C-reactive protein, iPTH: intact parathyroid hormone

Test	Result	Normal range
White blood count	4.70 × 10^9 ^/L	4-9 × 10^9 ^/L
Red blood count	3.17 × 10^12^ /L	3.80-5.10 × 10^12^ /L
Hemoglobin	9.7 g/dL	12.0-16.5 g/dL
Hematocrit	31.30%	35%-45%
Platelet count	1.31 × 10^11^ /L	1.5-3.5 × 10^11^ /L
Total protein	6.9 g/dL	6.7-8.3 g/dL
Albumin	3.6 g/dL	4-5 g/dL
Blood urea nitrogen	65.8 mg/dL	8-22 mg/dL
Creatinine	10.21 mg/dL	0.40-0.70 mg/dL
Uric acid	6.2 mg/dL	2.3-7.0 mg/dL
Sodium	137 mmol/L	138-146 mmol/L
Potassium	5.0 mmol/L	3.6-4.9 mmol/L
Chloride	98 mmol/L	99-109 mmol/L
Calcium	8.2 mg/dL	8.7-10.3 mg/dL
Inorganic phosphorus	6.9 mg/dL	2.5-4.7 mg/dL
Magnesium	2.2 mg/dL	1.8-2.4 mg/dL
Total bilirubin	0.4 mg/dL	0.3-1.2 mg/dL
AST	10 U/L	13-33 U/L
ALT	6 U/L	6-27 U/L
γ-GT	16 U/L	10-47 U/L
Creatinine kinase	35 U/L	45-163 U/L
CRP	0.36 mg/dL	<0.3 mg/dL
iPTH	2,356 pg/mL	10-65 pg/mL

Her iPTH level was markedly elevated, exceeding 30 times the upper limit of the reference range. Pre-dialysis serum inorganic phosphorus was elevated even for a dialysis patient, whereas corrected serum calcium remained within the normal range. Other laboratory results were unremarkable for a pre-dialysis patient.

**Figure 1:**
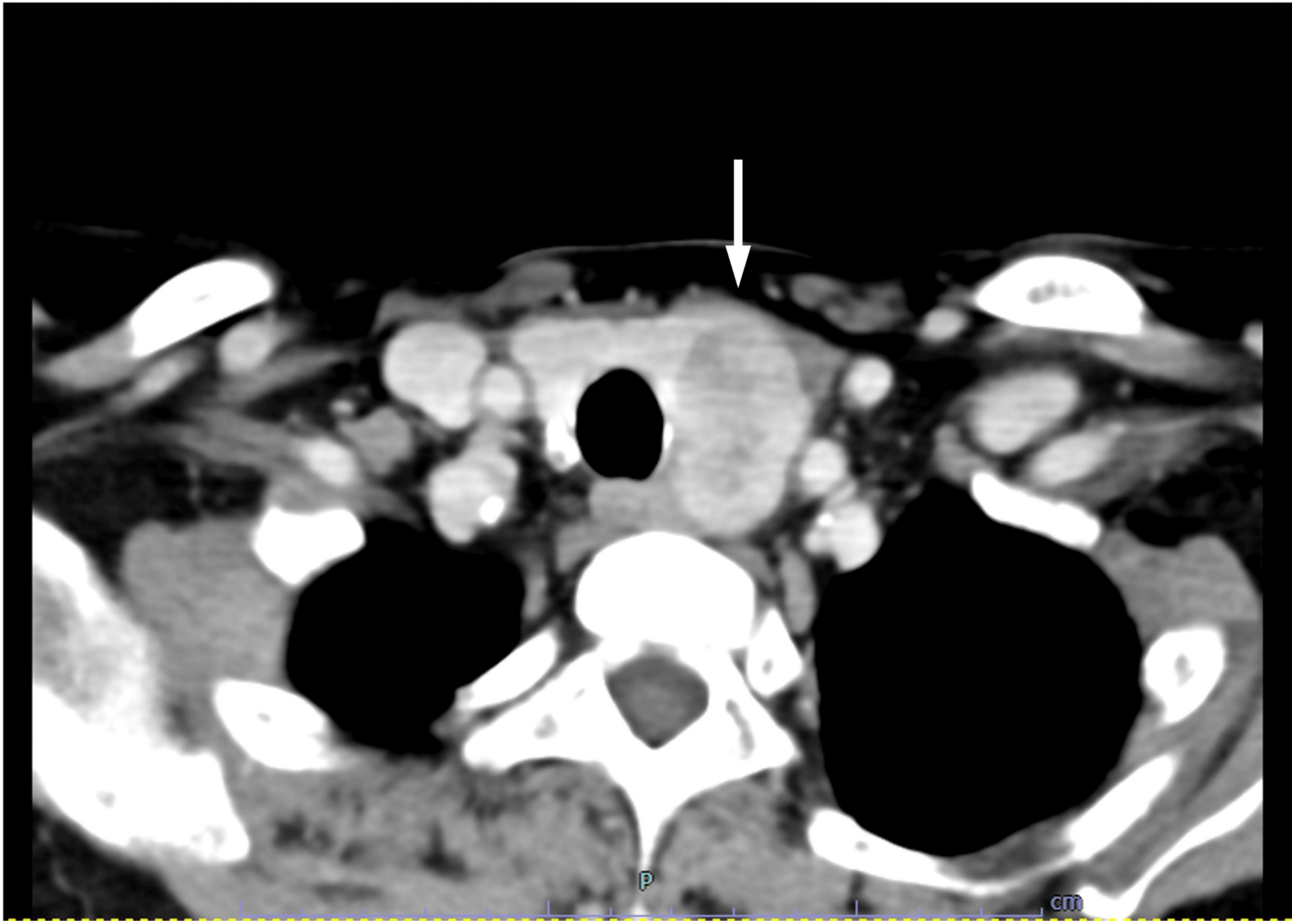
Horizontal slice of neck and chest contrast-enhanced CT scan A lobulated, low-attenuation mass measuring 33 × 21 mm was observed adjacent to the dorsal aspect of the left thyroid lobe (white arrow). Contrast enhancement was noted. No evidence of invasion into the surrounding tissues was observed. CT: computed tomography

Twelve-lead electrocardiography demonstrated QTc prolongation to 512 ms, with no other abnormalities. Chest radiography showed no cardiomegaly, and the costophrenic angles were sharp bilaterally. Non-contrast and contrast-enhanced computed tomography (CT) of the neck and chest demonstrated a 33 × 21 mm lobulated, low-attenuation mass located dorsal to the left thyroid lobe (Figure 1).

**Figure 2:**
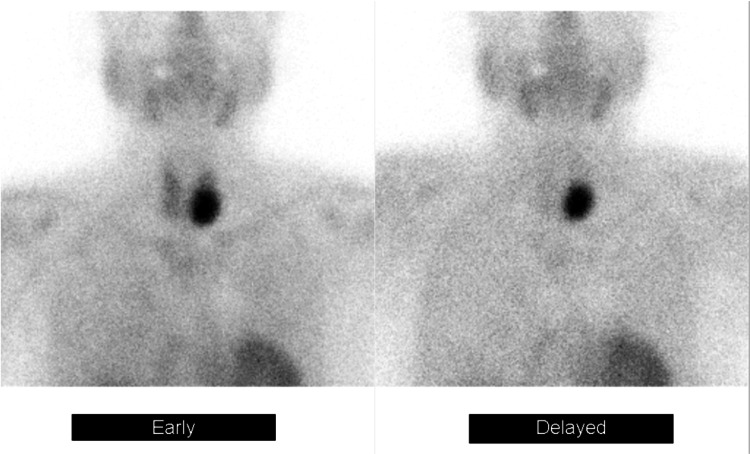
Tc-MIBI scintigraphy A nodular area demonstrating delayed uptake was observed near the left lobe of the thyroid. Tc-MIBI: technetium-99m methoxyisobutylisonitrile

The lesion exhibited contrast enhancement and contained small cystic degenerative changes, without evidence of invasion into adjacent structures. Tc-MIBI scintigraphy demonstrated a nodular lesion in the left thyroid lobe region with delayed tracer washout (Figure 2).

The patient was admitted for a planned parathyroidectomy. Considering that imaging studies revealed a solitary mass and laboratory findings suggested a functioning lesion, a parathyroid adenoma or malignancy of the left inferior parathyroid gland was suspected; however, given the markedly elevated iPTH level, surgical options included removal of the two left parathyroid glands or total parathyroidectomy (bilateral two-gland resection). After informed consent, the decision was made to remove only the left-sided glands (two glands) in this procedure. Because malignancy could not be excluded, a left thyroid lobectomy was also planned concurrently.

On hospital day 2, the patient underwent left parathyroidectomy (two glands) and left thyroid lobectomy. Two hours postoperatively, her corrected serum calcium level had decreased markedly to 7 mg/dL; continuous intravenous calcium gluconate hydrate infusion was initiated. As hypocalcemia persisted, the calcium gluconate dose was adjusted, and oral calcium supplementation was commenced. In light of postoperative hypophosphatemia, her pre-existing precipitated calcium carbonate was discontinued, and oral calcium lactate hydrate and vitamin D supplementation were initiated. Serum calcium and phosphorus levels subsequently stabilized within target ranges, and the patient was discharged home on postoperative day 8. Her iPTH decreased markedly to 18.2 pg/mL on the day after surgery and remained low thereafter (Table 2).

**Table 2:** Trends in corrected Ca, IP, and iPTH levels Ca: calcium, IP: inorganic phosphorus, iPTH: intact parathyroid hormone

Time points relative to the date of surgery	Six months earlier	Three months earlier	Preoperative	Postoperative day 1	Postoperative day 2
Ca (mg/dL)	8.8	8.6	8.6	8	8.2
IP (mg/dL)	9	8.4	6.9	4.3	3.6
iPTH (pg/mL)	1,980	2,512	2,356	18.2	21.1

Based on the histopathological findings of the surgical specimen demonstrating clear invasion of tumor cells into the thyroid tissue and positive immunostaining for parathyroid hormone (PTH), a diagnosis of parathyroid carcinoma was established.

**Figure 3:**
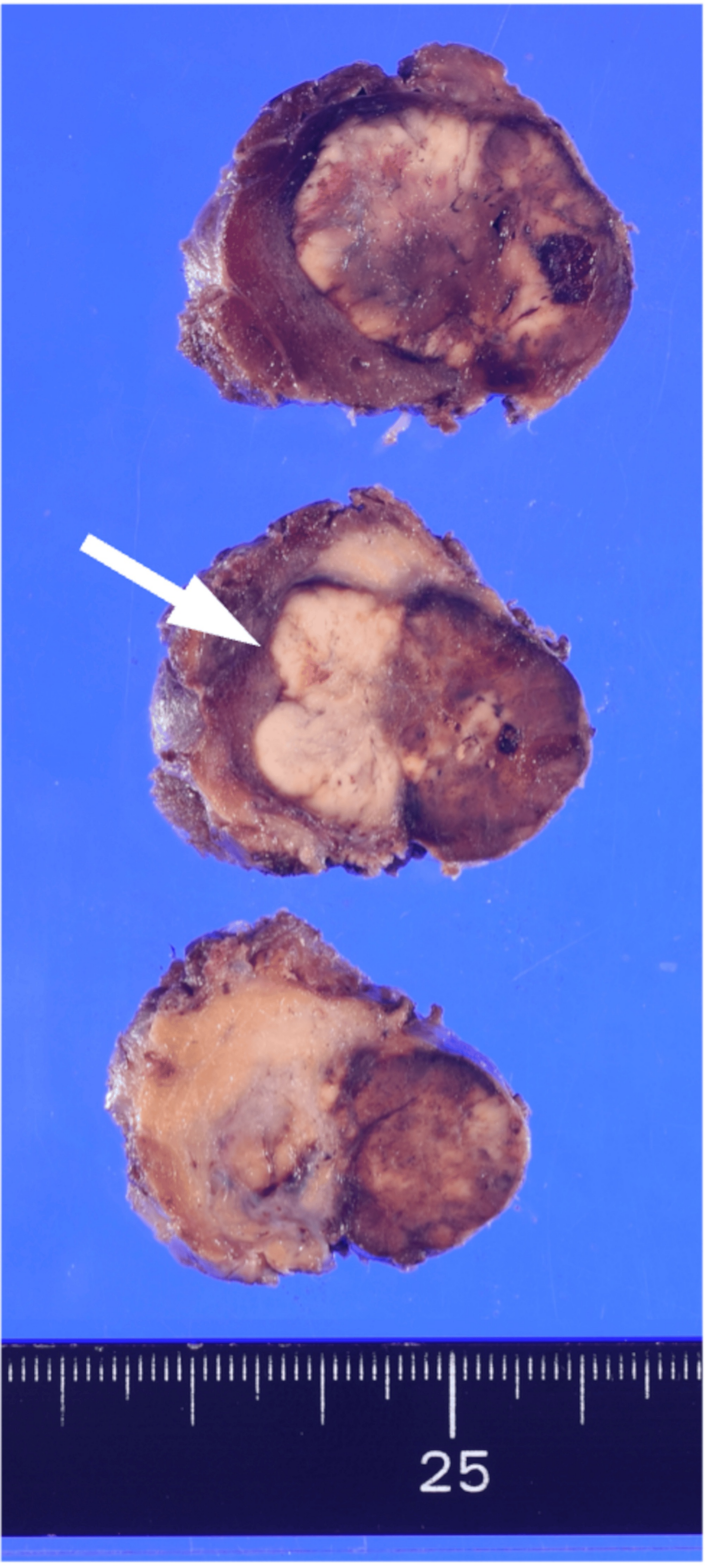
Surgical specimen (gross findings) The cut surface revealed a 25 × 25 mm white-to-brown mass invading the thyroid tissue. The white arrow indicates the approximate boundary between the thyroid (left) and the tumor (right).

**Figure 4:**
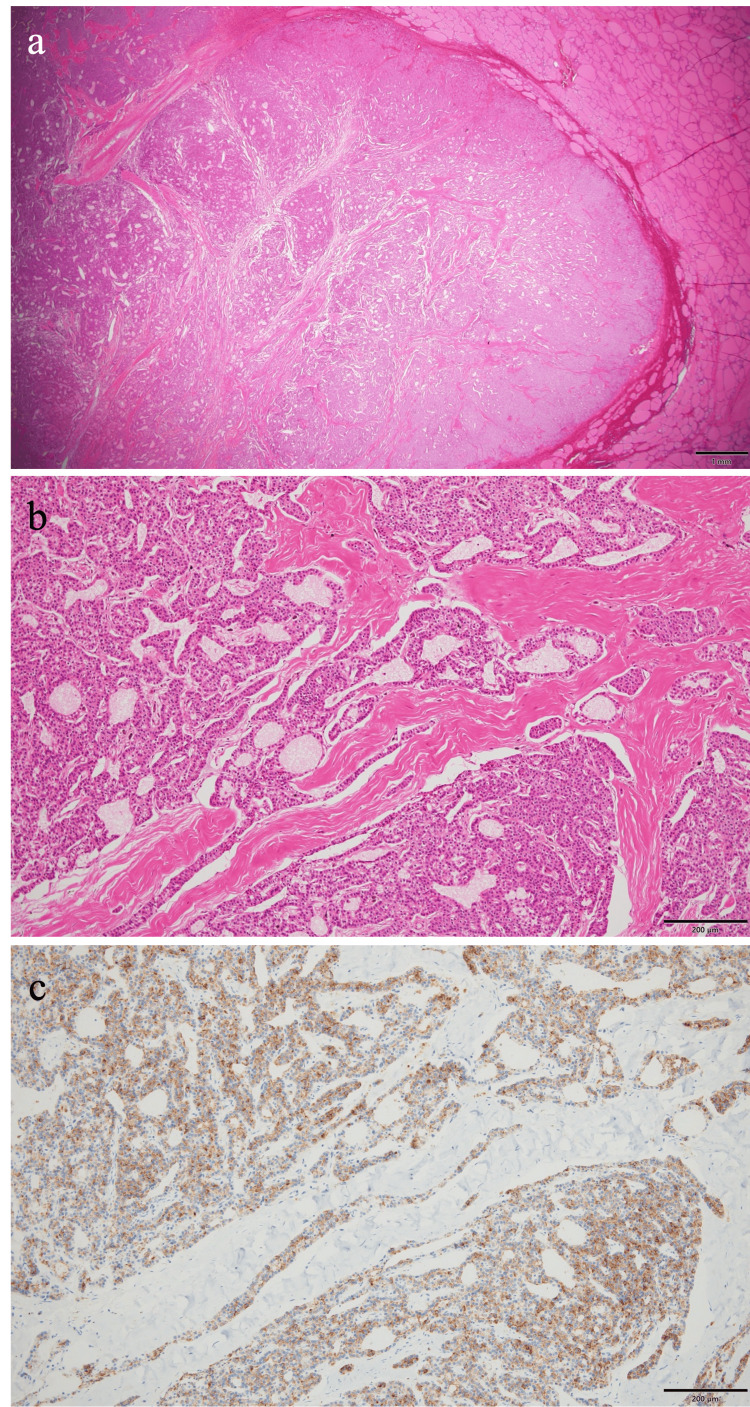
Surgical specimen (pathological findings) a: Low magnification, 12.5× (hematoxylin and eosin staining): invasion into the thyroid and adipose tissue was noted. b: High magnification, 100× (hematoxylin and eosin staining): the tumor consisted of chief cell-like cells proliferating in sheets or trabeculae. c: High magnification, 100× (PTH immunostaining): the tumor was positive for PTH. PTH: parathyroid hormone

The histopathological findings of the surgical specimen are shown in Figure 3 and Figure 4. Grossly, the cut surface revealed a 25 × 25 mm white-to-brown mass invading the thyroid tissue. Microscopically, the tumor consisted of chief cell-like cells proliferating in sheets or trabeculae. The tumor cells had clear to eosinophilic cytoplasm, and the nuclei were oval and mildly enlarged with minimal pleomorphism. Invasion into the thyroid and adipose tissue was noted. Vascular invasion was not evident. Immunohistochemically, the tumor was positive for PTH, negative for thyroid transcription factor 1 (TTF-1) and thyroglobulin, and had a Ki-67 labeling index of less than 1%.

## Discussion

We herein report a case of parathyroid carcinoma diagnosed following detailed evaluation and treatment of refractory hyperparathyroidism in a dialysis patient. The kidney plays a crucial role in bone mineral metabolism, and secondary hyperparathyroidism (SHPT) is recognized as a frequent and significant complication within the spectrum of chronic kidney disease-mineral and bone disorder (CKD-MBD), particularly in dialysis patients. While the high prevalence of SHPT among long-term dialysis patients is well established, reports of parathyroid carcinoma complicating dialysis patients are extremely rare [6,7]. Consequently, elevated intact parathyroid hormone (iPTH) levels in dialysis patients are often attributed to SHPT, potentially delaying the diagnosis of parathyroid carcinoma. Indeed, in the present case, the patient was initially referred to another hospital due to elevated iPTH levels; however, surgical intervention was not considered at that time.

Parathyroid carcinoma was first reported by de Quervain in 1904 as a nonfunctional metastatic carcinoma [8]. Even without limiting to patients on dialysis, parathyroid carcinoma remains an exceedingly rare disease. Its incidence among patients with primary hyperparathyroidism (PHPT) is estimated to be less than 1% [9]. Cases of parathyroid carcinoma complicating secondary hyperparathyroidism are even more exceedingly rare. Since the report by Berland et al. in 1982, it has been reported that only 37 cases have been documented in the English literature as of 2023 [6,10]. Reports of parathyroid carcinoma in patients undergoing dialysis for chronic renal failure are limited to only 34 cases in the English-language literature [11].

Globally, parathyroid carcinoma accounts for approximately 0.005% of all cancers [12]. While epidemiological data in Japan are limited, several international reports indicate an increasing incidence [13,14]. This trend may be attributable to improved diagnostic capabilities, the publication of guidelines for asymptomatic primary hyperparathyroidism in 2002, and an increase in surgical indications, leading to greater histopathological detection of parathyroid carcinoma [14].

The diagnosis of parathyroid carcinoma is usually confirmed by postoperative histopathological examination unless metastases are clearly present. Clinical features suggestive of parathyroid carcinoma include refractory hyperparathyroidism (especially iPTH levels exceeding three times the upper limit of normal), hypercalcemia (serum calcium levels ≥ 13-14 mg/dL), palpable cervical masses, and tumor size exceeding 3 cm [14-18]. In this case, although the preoperative serum calcium level was mildly low (Table 2), other clinical features were consistent with malignancy, indicating that these criteria were useful for predicting parathyroid carcinoma. Moreover, there are reports that serum calcium levels may be lowered by the use of calcimimetics even in the presence of parathyroid carcinoma, suggesting that the low serum calcium observed here could be attributed to etelcalcetide administration [19]. These findings suggest that the aforementioned features may be useful in predicting the likelihood of parathyroid carcinoma.

## Conclusions

We present a case of parathyroid carcinoma diagnosed as the cause of refractory hyperparathyroidism in a maintenance dialysis patient. While secondary hyperparathyroidism is highly prevalent in this population, it is essential to consider parathyroid carcinoma as a differential diagnosis, particularly when clinical features such as markedly elevated intact parathyroid hormone (iPTH) levels and large tumor size are present. An integrated approach that combines biochemical markers, imaging modalities, and clinical findings is essential for the early detection of parathyroid carcinoma in dialysis patients presenting with refractory hyperparathyroidism.
